# Electroencephalography-driven brain-network models for personalized interpretation and prediction of neural oscillations

**DOI:** 10.1016/j.clinph.2025.03.030

**Published:** 2025-06

**Authors:** Tena Dubcek, Debora Ledergerber, Jana Thomann, Giovanna Aiello, Marc Serra Garcia, Lukas Imbach, Rafael Polania

**Affiliations:** a Swiss Epilepsy Center, Klinik Lengg, Zurich, Switzerland; b ETH Zurich, Department of Health Sciences and Technology, Switzerland; c Neuroscience Center Zurich, University of Zurich and ETH Zurich, Switzerland; d AMOLF, Amsterdam, Netherlands

## Abstract

**Objective:**

Develop an encephalography (EEG)-driven method that integrates interpretability, predictiveness, and personalization to assess the dynamics of the brain network, with a focus on pathological conditions such as pharmacoresistant epilepsy.

**Methods:**

We propose a method to identify dominant coherent oscillations from EEG recordings. It relies on the Koopman operator theory to achieve individualized EEG prediction and electrophysiological interpretability. We extend it with concepts from adiabatic theory to address the nonstationary and noisy EEG signals.

**Results:**

By simultaneously capturing the local spectral and connectivity aspects of patient-specific oscillatory dynamics, we are able to clarify the underlying dynamical mechanism. We use it to construct the corresponding generative models of the brain network. We demonstrate the proposed approach on recordings of patients in status epilepticus.

**Conclusions:**

The proposed EEG-driven method opens new perspectives on integrating interpretability, predictiveness, and personalization within a unified framework. It provides a quantitative approach for assessing EEG recordings, crucial for understanding and modulating pathological brain activity.

**Significance:**

This work bridges theoretical neuroscience and clinical practice, offering a novel framework for understanding and predicting brain network dynamics. The resulting approach paves the way for data-driven insights into brain network mechanisms and the design of personalized neuromodulation therapies.

## Highlights

• Introduces new EEG-driven approach to identify dominant coherent brain oscillations.
• Integrates individualized model-based prediction of network dynamics and neurophysiological interpretability.
• Facilitates design of patient-specific neuromodulatory therapies in pharmacoresistant patients.

## 1 Introduction

Neural oscillations reflect information processing and communication in the brain, and patient-specific changes in oscillatory activity provide valuable insights into the state and progression of pathological brain activity (Buzsáki et al., 2013, Fries, 2015, Siegel et al., 2012). Oscillations arise from the local synchronization of neuronal firing, which allows cohesively incorporating meaningful information, but also from the synchronization of segregated brain areas, which ensures the temporal coordination of presynaptic and postsynaptic activation patterns in brain networks (Fries, 2015). Electroencephalography (EEG) is the most common method for monitoring neural oscillations and their changes over time, providing a non-invasive real-time window into brain dynamics (Niedermeyer’s Electroencephalography, 2017).

EEG analyses attempt to discover and interpret the activity patterns that characterize brain function and dysfunction. Over the years, they have facilitated significant advancements in understanding neural correlates of cognitive processes, diagnosing neurological disorders, and developing targeted treatments. Standard EEG analyses, however, face some challenges in the way to fully understand brain network dynamics: (i) They do not provide insight into the underlying dynamical mechanisms of the analyzed signals, nor how they could have been generated, but only describe patterns in the data. (ii) They assume stationarity, easily obscuring subtle but relevant changes in the inherently nonstationary nonlinear brain activity. (iii) They are easily influenced by noise, making it challenging to distinguish between consistently present coherent oscillations that carry meaningful information and accidental noisy contributions that are often not interpretable in the context of brain processes. (iv) They separately capture the local spectral and connectivity properties of dynamics, potentially missing critical insights into how these elements interact. For example, the most common approach used to determine individual alpha rhythms consists of finding the peak or”center of gravity” of the power spectral density in the *a priori* defined alpha frequency band (Corcoran et al., 2018).

A complementary approach are computational models, which attempt to simulate the brain electrical activity recorded by EEG measurements. EEG models provide a mathematical representation of the underlying dynamical mechanisms of the brain, allowing one to predict and potentially control the complex neural processes. This solves some of the challenges encountered in EEG analyses, but simultaneously introduces new ones: (i) They are often based on generalized assumptions about the brain and are not tailored to individual variations in brain structure and function, despite the recent recognition of personalized medicine. Modeling approaches mostly take two opposite paths: (ii) They mimic microscopic biological processes (Jirsa et al., 2017) and not effective emergent collective dynamics, thus becoming computationally intensive and impractical for largescale predictions or clinical use. (iii) Alternatively, they yield black-box data-driven models (Kerr and McFarlane, 2023, Wang et al., 2024), which can efficiently process large volumes of data, but are difficult to interpret in terms of neurophysiological processes, and thus hardly applicable by clinicians or neuroscientists.

All of these challenges highlight the need for a unification of patient-specific accuracy and neurophysiological interpretability. Without that, it remains difficult to fully understand and especially predict the complex network dynamics of healthy and pathological brain oscillations.

Among the main motivations for studying the mechanisms of brain oscillations is epilepsy (Devinsky et al., 2018)**.** Epilepsy is a neurological disorder characterized by recurrent seizures that stem specifically from abnormal (de)synchronization of neural oscillations in the brain network (Jiruska et al., 2013). It is one of the most common neurological disorders, affecting more than 50 million people worldwide (Devinsky et al., 2018), thus emphasizing the clinical relevance of understanding how brain oscillations can be well characterized, understood or modulated. Approximately a third of epilepsy cases are pharmacoresistant (Devinsky et al., 2018), leading to uncontrolled seizures and poor quality of life. This prompts the development of nonpharmacological approaches, such as epilepsy surgery (Zijlmans et al., 2019) or brain stimulation (Ryvlin et al., 2021, Xue et al., 2022). Epilepsy surgeries attempt to remove the part of the brain responsible for the pathological network oscillations. In 30 % of pharmacoresistant epilepsy patients, however, such a resection is not possible, e.g., if the identified area is also responsible for essential human functioning or is not unique. In these cases, therapies by brain stimulation are an option. They attempt to modulate collective neural dynamics via weak periodic varying currents (Johnson et al., 2020, Krause et al., 2023, Lozano et al., 2019, Riddle and Frohlich, 2021), ideally targeting critical nodes in the epileptic network and relevant oscillatory patterns, but much of their effect is still poorly understood. Hence, a good understanding of the oscillatory dynamics in the underlying epileptic network (Kramer and Cash, 2012, Li et al., 2021) and how these dynamics can be altered when they are pathological is needed. Furthermore, due to the heterogeneity of brain networks, especially in pharmacoresistant epilepsy patients, it is important to develop patient-specific approaches to the characterization and modeling of neural oscillations in the brain network.

The most severe brain state of pharmacoresistant epilepsy is refractory status epilepticus—a brain state of perpetual seizure activity (Pinto et al., 2022, Trinka et al., 2015). Status epilepticus is a medical emergency and, when refractory, has a mortality rate of 40 % (Pinto et al., 2022, Trinka et al., 2023), thus underscoring the need for novel therapy approaches. Remarkably, status epilepticus is a unique epileptic phenomenon also from a dynamical point of view: unlike most other epileptic seizures, which are characterized by a quick progression of dissimilar states of the epileptic network dynamics (Kramer & Cash, 2012), the brain activity in status epilepticus can be well described as steady-state dynamics (Burman et al., 2020)**.** Dynamics that are steady-state can be characterized by quantities that are relatively stable in time and the equations describing them are often more interpretable. Therefore, status epilepticus is an excellent point of departure for the development of data-driven predictive analyses and models that could help the progress of emerging therapeutic procedures. Namely, once the dynamics in the (pathological) steady state are understood and captured, an immediate model-based search for the external influence that restores the dynamics of another (healthy) steady state becomes possible.

**Fig. 1:**
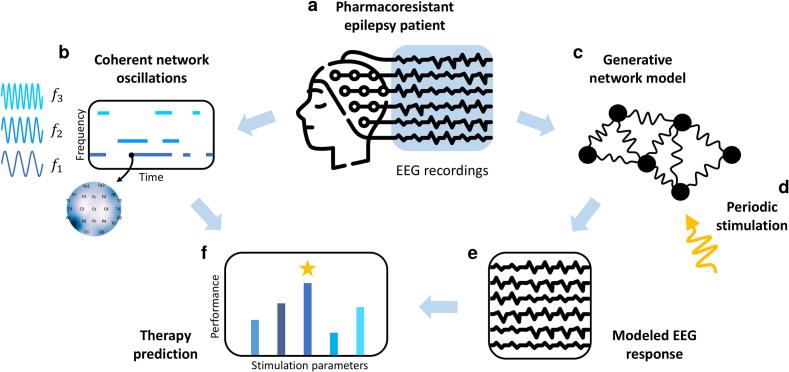
Personalized EEG-driven modeling of epileptic network dynamics and their modulation — **a** EEG recordings of (pharmacoresistant) pathological brain activity can be used to **b** identify the dominant coherent neural oscillations and **c** construct generative models of the underlying epileptic network dynamics. **d** The effective dynamical connectivity matrix of the epileptic network is encoded in the models and can predict the network dynamical reaction to an external intervention, e.g., brain stimulation. **e** The proposed fully data-driven paradigm can help understand the response to interventions and **f** anticipate the performance of new therapies.

Here, we introduce a procedure to identify the dominant coherent neural oscillations (Fig. 1b) and constructing corresponding personalized oscillator-network models (Fig. 1c) of epileptic network dynamics and apply it to recordings of patients in status epilepticus (Fig. 1a). The procedure leverages Koopman operator theory (Brunton et al., 2016a, Brunton et al., 2021), integrating personalization and precision with neurophysiological interpretability and computational feasibility. Additionally, it employs adiabatic theory to adapt contemporary Koopman algorithms to the inherently non-stationary and noisy EEG dynamics. The extracted coherent neural oscillations and corresponding models are fully data-driven and do not rely on predefined assumptions, thus being tailored to individual patients, and simultaneously provide interpretable representations of the epileptic network dynamics within a conventional neurophysiological framework. Furthermore, the extracted models are generative, yielding an effective dynamical connectivity matrix that reflects the characteristics of the individual network and allows for the prediction of unseen scenarios, such as the network response to external interventions (Fig. 1d, e, f).

## 2 Methods

### 2.1 EEG recordings and preprocessing

We considered electroencephalographic (EEG) recordings of 10 patients in nonconvulsive status epilepticus (Fig. 1a). In 5 of the cases, the status was resolved after the administration of pharmacological therapy. The brain state with ongoing status epilepticus was termed Status, and the brain state after its resolution was termed Resolved. The EEG recordings were recorded on a clinical routine EEG device (*Nihon Kohden*), with a 10–20 EEG system and a sampling rate of 200 Hz. Since recordings of non-convulsive status epilepticus are minimally affected by artifacts, we only high-pass-filtered the EEG signals at 0*.*5 Hz. Furthermore, since we were mainly interested in the lower brain bands, we resampled the signals to 100 Hz. This was motivated by the steep increase in the computational cost of modeling with the sampling rate. The local ethics committee approved the study and all patients or legal representatives gave their informed consent. However, this consent did not include a provision stating that individual data can be made freely accessible.

### 2.2 Modeling of network dynamics via Koopman theory

The dynamics of the epileptic network, monitored by EEG measurements, is an instance of complex nonlinear dynamics. Due to the complexity of the brain and the presence of various types of noise, it cannot be represented by any means through a simple set of equations. An optimal approach for modeling such dynamics, thus, has to be significantly data-driven. If interested in a data-driven yet interpretable modeling paradigm for complex nonlinear systems, Koopman analysis emerges as an excellent basis (S. L. Brunton et al., 2016a, Brunton et al., 2021).

Following Koopman operator theory (S. L. Brunton et al., 2021), the nonlinear dynamics of the epileptic network $x_{t}$ can be expressed, through a suitable coordinate transformation *g*, as linear dynamics governed by the infinite-dimensional Koopman operator *κ*,

$$g\left(x_{t+1}\right)=\kappa g\left(x_{t}\right).$$

Here *x_t_* is a vector containing the EEG measurements in all channels at time *t*. The eigenvalue decomposition of the Koopman operator *κ* yields the Koopman modes, which capture distinct spatio-temporal patterns of the epileptic network.

The goal of related algorithms is then to find a tractable finite-dimensional linear representation of *κ*. One of the most reliable algorithms is based on the so-called Dynamic Mode Decomposition (DMD) (Schmid, 2010). It seeks the best fit linear operator *A* that approximately advances the state of the discrete-time system *x_t_* forward in time, to *x_t_*_+1_,

$$x_{t+1}=Ax_{t},$$

The operator *A* is obtained through a high-dimensional linear regression of the dynamics that evolve the matrix of consecutively measured states *X*,

$$X=\left[\begin{array}{ccc} | & |\\ x_{0} & x_{1} & \cdots \\ | & | \end{array}\begin{array}{c} |\\ x_{m-1}\\ | \end{array}\right],$$

for one time step, to X′=AX,

(4) $$X^{\prime }=\left[\begin{array}{ccc} | & |\\ x_{1} & x_{2} & \cdots \\ | & | \end{array}\begin{array}{c} |\\ x_{m}\\ | \end{array}\right].$$

The DMD algorithm yields the so called DMD modes, which are related to *A* through the eigenvalue decomposition,

(5) A=ΦΛΦ†,

where Φ is the matrix of DMD eigenvectors, and Λ the diagonal matrix of corresponding DMD eigenvalues. DMD modes can be interpreted as Koopman modes and correspond to the dominant spatially coherent oscillations, whose frequency and growth/decay rate are determined by Λ, and whose spatial profile is determined by Φ.

The DMD algorithm (S. L. Brunton et al., 2021) is based on singular value decomposition (SVD), thus avoiding stochastic and heuristic optimization and allowing one to find solutions even when the high-dimensional landscape of solutions is complex and/or nonconvex. It was originally introduced in the fluid dynamics community and has to be adapted for electrophysiological recordings (Brunton et al., 2016a). That is, matrices *X* and *X*′ have to be augmented by (vertically) stacking time-shifted copies of the original data matrices. The choice of the temporal (horizontal) dimension of the matrices *X* is determined by the choice of the sliding time window to which the DMD algorithm is applied. However, the maximal number of eigenmodes (and DMD modes) is determined by the smaller of the two dimensions of the matrices *X*. Due to dense time sampling and sparse spatial sampling, this number is typically confined by the number of channels in the EEG recordings. Constructing augmented data matrices, thus, allows to obtain enough DMD modes to capture the considered dynamics. Using augmented data matrices is justified as long as all corresponding time-shifted copies of data are close enough that their dynamics can be described by the same operator *A*.

For a broader overview of Koopman theory and for more details on our application of it to epilepsy recordings, see SI.

### 2.3 Extraction of adiabatically evolving coherent oscillations

The DMD algorithm identifies the dominant coherent oscillatory modes in a chosen time window. However, only some of the identified DMD modes survive through longer time scales. To identify the most persistent coherent oscillations in the epileptic network, we followed the evolution of DMD modes and identified those whose change in frequency $\Delta f=f_{t+1}-f_{t}=\left(\omega_{t+1}-\omega_{t}\right)/\left(2\pi \right)$ and evolution speed of complex eigenvectors *o* = 〈*φ_t_*_+1_|*φ_t_*〉 are slow enough, i.e., the most stable modes.

Once we identified the most stable DMD modes, they were used to define the effective long continuous oscillatory modes in the epileptic network (Fig. 1b). Their effective frequencies were given by the peaks in the frequency distributions. Their effective amplitudes and phases were given by the peaks in the corresponding joint density function. The density function was estimated nonparametrically from the set of DMD modes for all time windows, using Gaussian kernel density estimation (implemented by Python *stats* library).

For more details on the algorithm behind the identification of personalized persistent oscillations in the epileptic network, see below.

#### 2.3.1 Stable coherent oscillations from DMD modes

The DMD algorithm identifies the dominant coherent oscillatory modes in the time window defined by the data matrices considered. However, the length of these time windows is restricted: Considering a very long time window means further increasing *n*, which then requires high stacking degrees *h* to avoid a strong mismatch of the two matrix dimensions (see above). Having high stacking degrees in the data matrix, in return, implicitly assumes that the dynamics through a fairly long time is steady enough to be best approximated by the same choice of operator A—an assumption that is easily invalidated when considering EEG recordings. We therefore chose the minimal time window length *n* that is enough to capture all frequencies of interest, i.e., *n* = 200 corresponding to 1 s (see below). For each time window, we extracted the single-time-window (*instantaneous*) coherent modes by the DMD algorithm applied to the augmented data matrices.

The DMD modes inform about the dominant coherent oscillations in the epileptic network in one of the time windows of chosen length *n*. However, not all identified DMD modes necessarily reflect the oscillations that survive through longer time scales, as it can happen that a short accidental noise contribution dominates the stable brain oscillations in one time window. In order to identify the most persistent and stable coherent oscillations in the epileptic network, one needs to consider which of the DMD coherent modes continuously arise as the DMD window slides through time. Therefore, we followed the evolution of the spatial and spectral profile of the DMD modes (determined by the DMD eigenvectors and eigenvalues, respectively), and only kept those modes that change slowly enough, *adiabatically*. The evolution speed of the eigenvalues for a stable system $(|\lambda |=|e^{i\omega }|=1$) can be quantified by the change in their frequency $\Delta f=f_{t+1}-f_{t}=\left(\omega_{t+1}-\omega_{t}\right)/\left(2\pi \right)$, while the evolution speed of the complex eigenvectors is quantified by their Hermitian scalar product $o=\langle \varphi_{t+1}|\varphi_{t}\rangle$. For the choice of Δf and o hyperparameters, see SI.

#### 2.3.2 Effective eigenmodes via Gaussian kernel density estimation

Once the most stable DMD modes were identified, we used them to define the effective long continuous oscillatory modes in the epileptic network. The effective frequencies of the dominant network oscillatory modes were signaled by the peaks of the frequency distributions (Fig. 1a,b, right panels). The effective spatial profiles (amplitudes and phases) were obtained from the distributions of all identified long continuous oscillatory modes. The spatial properties of each DMD mode are encoded in a complex eigenvector

$$\phi ={\left[\phi^{\left(0\right)},\phi^{\left(1\right)},\cdots ,\phi^{{(n}_{\mathrm{ch}}-1)}\right]}^{T}$$

$$={\left[a^{\left(0\right)}{e^{\varphi }}^{\left(0\right)},a^{\left(1\right)}{e^{\varphi }}^{\left(1\right)},\cdots ,a^{\left(n_{\mathrm{ch}}-1\right)}{e^{\varphi }}^{\left(n_{\mathrm{ch}}-1\right)}\right]}^{T},$$

where *n_ch_* is the total number of EEG channels, {*a*^(^*^j^*^)^} is the corresponding set of complex amplitudes and {*φ*^(^*^j^*^)^} is the corresponding set of complex phases. We used Gaussian kernel density estimation (implemented by Python *stats* library) to nonparametrically estimate the joint density function of complex amplitudes {*a*^(^*^j^*^)^} and phases {*φ*^(^*^j^*^)^} for all stable coherent oscillations. The maxima of these density functions were then used to define the patient-specific effective amplitudes and phases of the dominant coherent oscillations.

### 2.4 Model extraction and validation

The DMD eigenvalues and eigenvectors, and the corresponding dynamical connectivity matrices *A*, were used to define the model of epileptic network dynamics for each time window. In addition, a Broyden–Fletcher–Goldfarb–Shanno (BFGS) algorithm was applied to find the boundary conditions that optimally reproduced the local spectral properties of the measured recordings.

The reliability of EEG-driven models was systematically evaluated using the structural similarity index (SSIM) and classification metrics, including accuracy and receiver operating characteristic (ROC) area under the curve (AUC).

#### 2.4.1 Structural similarity index (SSIM)

The SSIM was used to assess the local spectral and connectivity properties of measured and modeled EEG data. For spectral analysis, SSIM was calculated for the power spectral density (PSD) matrices of the two states (Status and Resolved) across all combinations of measured and modeled data. For connectivity analysis, SSIM was calculated for the debiased weighted phase lag index (dwPLI) matrices in all frequency bands. The SSIM quantifies the similarity between two matrices, with a value of 1 indicating identical properties. To evaluate statistical significance, the p-values for each SSIM element were computed using channel-wise permutation testing. For each pairwise comparison, the labels were shuffled N=1000 times, generating a null distribution of SSIM values under random conditions. The p-value for a given SSIM element was defined as the proportion of permuted SSIM values that exceeded or equaled the observed SSIM. Elements with p≤0.05/6 (Bonferroni-corrected for 6 pairwise comparisons across measured and modeled data in Status and Resolved) were retained, while nonsignificant elements were masked in the SSIM plots.

#### 2.4.2 Classification metrics

Binary logistic regression was used to classify PSD and dwPLI matrices into Status and Resolved states. The model was trained and tested on all combinations of measured and modeled data (*train measured test measured, train measured test modeled, train modeled test measured, train modeled test modeled*), ensuring comprehensive evaluation of classification performance. Accuracy was calculated as the proportion of correctly classified samples, reflecting the classifier’s ability to differentiate between the two states. ROC-AUC was computed to quantify the classifier’s ability to rank predictions correctly. The classification metrics were derived using 10-fold cross-validation for each train-test combination, ensuring the robustness of the results. Confidence intervals were computed from N = 10000 bootstrapped distributions of these metrics. We note that the SSIM-based connectivity accuracy evaluation was performed on dwPLI matrices, which inherently combine values across all epochs during the debiasing process. In contrast, the classifier-based statistical validation utilized wPLI matrices without debiasing, allowing distributions to be derived from individual epochs.

### 2.5 Modeling and analysis hyperparameters

The values of certain hyperparameters in the modeling and analysis procedures were fixed on the basis of the existing literature and / or explicit testing. This includes resampling frequency and other scales when performing preprocessing of raw signals, epoching, performing dynamic mode decomposition, extracting continuous coherent oscillations and optimizing models. For the specific chosen values of these hyperparameters, see SI.

## 3 Results

### 3.1 Identification of robust coherent oscillations

Local spectral methods such as electrode-specific power spectral density give insights into the power frequency dependence of certain dynamics, but do not inform how coherent that power is. Connectivity properties, on the other hand, are of great importance when considering the dynamics of the epileptic network and the oscillations therein, motivating the development of numerous connectivity and interaction measures to include in EEG analyses (Cliff et al., 2023). However, extracting an interpretable model of dynamics from metrics of local power and connectivity has remained a challenge.

The method we developed identifies the most persistent coherent oscillations directly from EEG data recorded at a certain point in time (Fig. 1a). It is based on a combination of an extended dynamical mode decomposition (DMD) algorithm (Brunton et al., 2016a; S. L. Brunton et al., 2021), an instance of Koopman operator theory (S. L. Brunton et al., 2021) and an adaptation of the theory of adiabatic evolution (Joye & Pfister, 1994) (see Methods). Coherent oscillations, automatically identified by the DMD algorithm, are followed through time via a sliding window, and only oscillations whose frequency and spatial profiles are stable are kept as relevant (for more details of the basic algorithms and reasoning, see Methods). In contrast to standard spectral estimates, the method discriminates between robust coherent oscillations in the EEG dynamics and accidental noisy contributions to its power spectrum, so that fundamental brain oscillations can be recognized (Fig. 1b). Their properties and dynamical interactions are obtained fully data-driven and can serve to build up a patient-specific interpretable model of the core EEG dynamics (Fig. 1c), and could be used to model its interaction with an external stimulation source (Fig. 1d-f).

**Fig. 2:**
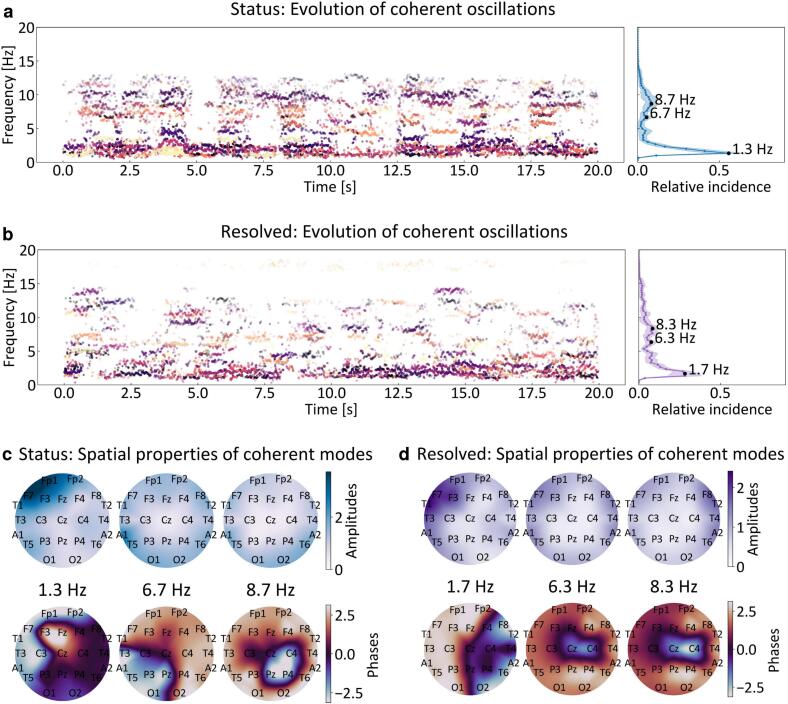
Robust coherent oscillations in the epileptic network — By identifying the most persistently coherent oscillations directly from the recorded EEG data, our method discriminates the fundamental brain oscillations from accidental noisy contributions to the power spectrum. **a** Example of time evolution of the robust coherent oscillations in one 20 s long epoch of status epilepticus recordings (left), and the normalized frequency distribution of their incidence in all considered epochs (right) for patient labeled as EEG 3. The different colors in the time evolution correspond to different coherent oscillatory modes, whereas the opaqueness is related to their strength. The peaks in the incidence distribution indicate frequencies at which pronounced coherent oscillations in the epileptic network are present. The line denotes the frequency-specific time-wise average of the oscillation incidence, the shaded area denotes the corresponding standard deviation. **b** Analogous results for the recordings after status epilepticus is resolved. The relative incidence of very low frequency coherent oscillations is reduced, and several peaks at higher frequencies appear. **c**, **d** Spatial profiles (distributions of amplitudes and phases) of the identified most prominent coherent oscillations for both states. The amplitude distributions indicate the EEG locations at which the strong coherent dynamics is most prominent. The phase distributions inform whether the coherent oscillations in different brain regions are fully in phase (homogeneous color) or a consistent phase shift is present (change of color).

We extracted the time evolution of the robust coherent oscillations for each patient and each brain state (Fig. 2a,b, see SI). The frequency distributions of the relative incidence of coherent oscillations in all epochs (Fig. 2a,b, right panels) were used to determine the frequencies at which pronounced coherent oscillations are present in the epileptic network. As intuitively expected in status epilepticus, characterized by pronounced slow activity and epileptiform discharges (Trinka & Leitinger, 2015), the relative incidence of coherent modes had a significantly higher peak at very low frequencies (*δ*-band). Smaller local distribution maxima at frequencies higher than the *α*-band appeared only after the pathological state was resolved.

After determining the frequency of the pronounced coherent oscillations, we analyzed their spatial profiles, that is, the corresponding distribution of amplitudes and phases on the scalp (Fig. 2c,d). The high-amplitude regions in the amplitude distributions were used to detect regions in which the EEG dynamics consistently have strongly coherent oscillations. The phase distributions informed about the relative phase of the ongoing strong oscillatory dynamics. Namely, strongly coherent oscillations at a certain frequency can still have a consistent phase shift: In the topographic maps of phases, zero-phase synchronized dynamics are reflected in homogeneously colored scalp regions, while changes in color are related to permanent phase shifts (only a relative difference is meaningful in the context of phases). These phase shifts might be of crucial importance when trying to interact with the oscillating network by (resonant) stimulation.

### 3.2 Generative coupled-oscillator model of network dynamics

**Fig. 3:**
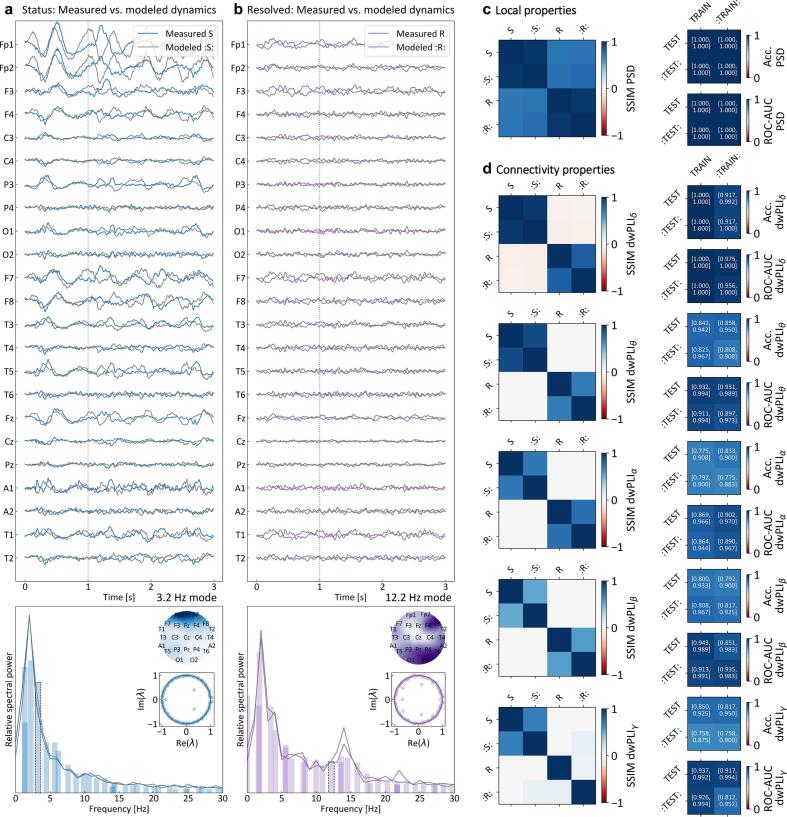
Coupled-oscillator model of epileptic network dynamics — The identified EEG-based coherent oscillations can be used to define a generative effective model of the brain dynamics. The data-driven model is constructed so to capture the clinically relevant properties of EEG dynamics: spectral content, inter-regional synchronization, amplitude variation between different channels and states. **a**, **b** Examples of compared measured and corresponding modeled EEG dynamics in the two states (top). The effective model was extracted from the first third of the shown dynamics (left of vertical dashed line), while its generative nature and steadiness of the modeled states were exploited to generate the rest. The frequencies and relative powers of the widespread oscillatory modes building up the model follow the corresponding spectral power distribution (bottom). Each of the modes has a defined spatial distribution. The inset plots show examples of such distributions (inset topo-plots) and stability exponents (real vs. imaginary components of eigenvalues *λ*) for one of the modes in each state (3.2 Hz in Status, as 12.2 Hz in Resolved). **c***Left* Time-averaged structural similarity indices (SSIM) of measured and modeled power spectral density (PSD) for all combinations of the two states (see **a, b** for label interpretation). The block-diagonal structure of the SSIM matrices reflects the model’s ability to replicate local spectral differences and similarities between the states. *Right* Statistical validation using binary logistic regression classification of PSD matrices for Status and Resolved recordings, with confidence intervals (CI) of accuracy and receiver-operating-characteristic areas (ROC-AUC) shown for all train-test combinations (training or testing on modeled recordings is denoted by dotted labels). Perfect classification and high ROC-AUC confirm the model’s fidelity. **d***Left* Time-averaged structural similarity indices (SSIM) for the debiased weighted phase lag index (dwPLI) in five frequency bands, comparing measured and modeled connectivity across states. The SSIM matrices show a mostly block-diagonal structure in the lower three bands (*δ*, *θ*, *α*), reflecting the model’s ability to capture connectivity patterns in low-frequency dynamics. In higher bands (*β*, *γ*), the structure becomes less defined, indicating weaker coherence of measured data. White fields represent combinations with non-significant SSIM results (*p >* 0*.*05/6). *Right* Binary logistic regression results (CI of accuracy and receiver-operating-curve area ROC-AUC) for classifying dwPLI matrices between Status and Resolved recordings. Accuracy decreases only slightly in higher frequency bands and ROC-AUC remains close to 1, indicating robust state differentiation by the model.

Our next goal was to construct a corresponding effective individual model of brain dynamics (Fig. 1c). For each sliding time window, we extracted a model that consists of coupled oscillators, algorithmically identified for each specific case via the extended DMD procedure (Fig. 1c, see Methods). The generative model reproduced the EEG dynamics with the correct properties, including local spectral content, interregional synchronization, and amplitude variation (Fig. 3a, b). Crucially, due to its oscillatory nature and the steady-state property of the data, the dynamics generated by the model fitted on a certain window could also be extrapolated to later times (Fig. 3a, b, the fitting and extrapolation region are separated by vertical dashed lines). Precisely due to this property, the model could later be used to probe the network response under an external influence (Fig. 1d).

To systematically evaluate the reliability of EEG-driven models, we compared standardized measures of local spectral and connectivity properties of the EEG dynamics in all pairs of measured and modeled data in the two states. The local spectral agreement was evaluated using the time-averaged structural similarity index (SSIM) of the power spectral density (PSD) (Fig. 3c, see Methods and SI). The resulting SSIM matrix has a clear block-diagonal structure, with very strong similarity (SSIM PSD *>* 0*.*9) within- and significantly weaker similarity (SSIM PSD *<* 0*.*5) between-states (approximately uniform color of the two offdiagonal blocks of the SSIM matrix in Fig. 3c, left). Intuitively, it means that the PSD matrices of different states have a lower level of similarity, and this is well reproduced by the model. Further validation using a binary logistic regression classifier on epoch-based PSD matrices, evaluated across all combinations of measured and modeled data, yielded perfect accuracy and ROC-AUC values of 1, confirming that both measured and modeled PSDs equally well differentiate between the two states (Fig. 3c, right).

The reliability of the modeled connectivity properties was evaluated using the SSIM of the debiased weighted phase lag index (dwPLI) in five frequency bands (Fig. 3d, left). The lower bands (*δ*, *θ*, *α*) exhibited a mostly block-diagonal structure, reflecting coherent connectivity patterns effectively captured by the model. Higher bands (*β*, *γ*) showed a less pronounced block-diagonal structure, reflecting the loss of clear coherent oscillations that could be targeted by the model. Despite this, the consistently high accuracy and ROCAUC values from the classification of epoch-based weighted phase lag indices (Fig. 3d, right) demonstrated that the model successfully captures and differentiates relevant connectivity patterns in all meaningful frequency domains.

## 4 Discussion

In this work, we aimed to develop personalized EEG-based methodologies to advance understanding and prediction of brain dynamics. We demonstrated the utility of these methods by applying them to recordings of status epilepticus—a life threatening condition of continuous seizure activity that underscores the need for deeper insights and innovative therapeutic approaches. By integrating EEG analyses with computational models, our approach allows for a data-driven characterization of brain dynamics, which not only quantifies the local spectral and connectivity properties of neural oscillations, but also provides a deeper insight into the dynamical mechanisms underlying these oscillations. Such duality holds the potential to enable tailored therapeutic strategies that are both patient-specific and interpretable within existing clinical and neuroscientific frameworks.

The heterogeneity of epilepsy, in terms of semiology, etiology, phenotypes, and genotypes, is remarkable. However, many of the differences have been overlooked within standard clinical and research approaches to diagnosis and treatment. Only recently,’personalized medicine’ slowly started entering the field of epilepsy and introduced data-driven tailoring of pharmacological and surgical treatments (Jehi, 2023, Jirsa et al., 2017, Lhatoo et al., 2020, Nabbout and Kuchenbuch, 2020, 2020; Steriade, 2022). New patient specific treatments and methods are developed, motivated by the high percentage of pharmacologically uncontrolled epilepsies (Devinsky et al., 2018). Yet, as epilepsy patients are a vulnerable population, they underline the need for a careful approach to new methods and technologies. One aims for approaches that unify personalized quantitative characterization and prediction of brain dynamics, with interpretability in the context of standard clinical and neuroscientific theories. Namely, interpretability in the context neural oscillations (Engel et al., 2001, Fries, 2015, Keitel et al., 2022), which spread through the brain network, which are pathologically altered in cases of disease, and which are modulated by novel therapies.

Brain dynamics are typically characterized by EEG analyses that accurately quantify the local spectral and connectivity properties of network dynamics (Das et al., 2023). These analyses are fully personalized in the sense that their results are fully EEG-driven. However, they do not provide an intuition about the responsible dynamical mechanisms in the brain network. Most of our understanding of brain dynamics and of new therapies to modulate them is, on the other hand, based on effective models of neutral oscillations and their dynamical mechanisms. For example, the role of theta oscillation in epilepsy (Roliz & Kothare, 2023) or oscillation entrainment in transcranial alternating current stimulation (tACS) (Johnson et al., 2020, Krause et al., 2023, Liu et al., 2018)**.** Standard EEG analyses do not always offer a clear link between personalized results and the dynamics they should ideally characterize or predict, that is, they are often not easy to interpret in terms of standard clinical or neuroscientific theories.

In the first part of our work, we showed that the characterization of local spectral and connectivity properties can be done to simultaneously yield the corresponding neural oscillations in the brain network and provide a clear path towards a comprehensive dynamical model of the underlying network. Being fully data-driven and based directly on EEG recordings, the algorithm allowed us to fully circumvent generalized assumptions about anatomical and functional connections, and retain an objective quantitative picture of the network dynamics. By algorithmically discriminating between robust coherent oscillations and accidental noisy contributions to the power spectrum, we were able to recognize patient- and state-specific fundamental brain oscillations.

When personalized approaches are needed, brain dynamics are typically modeled by machine

learning (ML) models (Kerr and McFarlane, 2023, Wang et al., 2024)**.** ML models can yield precise approximations of the modeled dynamics and predictions about it, but mostly of a”black-box” type and without clear interpretation. The lack of interpretability is a common problem for ML models also in other domains of technology and society (Adadi and Berrada, 2018, Jirsa et al., 2017). This makes their use difficult in the clinical setting, where transparency and reliability are crucial. A viable alternative are biologically motivated models (Jirsa et al., 2017). However, because of the microscopical details they are built on, (i) they tend to be computationally expensive, and (ii) they do not have an easy connection to the more macroscopic oscillation-based clinical picture. These aspects lead to significant challenges for their implementation in clinical settings.

In the second part of our work, we showed how to build data-driven models of the brain network dynamics that simultaneously (i) accurately reproduce the personalized clinically relevant properties of EEG dynamics (spectral context, inter-regional synchronization and amplitude variation between different channels and states), and (ii) yield an effective equation of motion (encoded in a dynamical connectivity matrix) for the brain network oscillations and thus an interpretation in terms of existing neural oscillation theories.

Before closing, we highlight the limitations and potential expansions for the clinical application of the presented methods. The reliance on Koopman operator theory provides an effective linear representation of instantaneous dynamics, while adiabatic tracking assumes that slowly evolving modes can be locally characterized by a single frequency. However, this approach may not fully capture complex temporal structures arising from intracerebral and sensory-motor interactions, or externally stimulated nonlinear dynamics. Future directions could involve incorporating recent extensions of Koopman algorithms that explicitly include nonlinear terms (Brunton et al., 2016b, 2021; Kalur et al., 2023), utilizing measures such as the Higuchi fractal dimension (Higuchi, 1988) for local dynamics and the Frechet or normalized compression distance (Bennett et al., 1998, Fréchet, 1957) for connectivity dynamics, and tracking oscillatory modes using more sophisticated temporal characterizations. In our current work, the model was validated using internal patient datasets, which may not fully capture the variability present in external populations. Future work should aim to test the model on independent datasets and thus further assess its robustness and generalizability. Furthermore, the proposed modeling paradigm is currently computationally demanding, with about a day needed to obtain the results for a specific dataset. However, optimizing speed was beyond the scope of this work. Notably, the method is inherently parallelizable, as sequential time windows can be processed independently, making it well-suited for efficient execution on modern computing infrastructure. Future work should focus on enhancing computational efficiency through parallelization, improved integration of clinical and computational hardware, and optimized modeling hyperparameters to enable real-time clinical applications.

In terms of clinical applications, two promising directions emerge. The first involves conditions in which the brain network states gradually evolve, such as the spread and propagation of coherent oscillations in focal epilepsy. In these cases, the corresponding EEG dynamics cannot be approximated as steady state. Instead, the presented methods could identify robust coherent oscillations to quantitatively assess the evolving dynamics of the brain network. For example, in the presurgical evaluation of epilepsy patients with invasive recordings, this approach could model seizure-related dynamics both within the seizure onset zone and across large-scale networks. By tracking the spatial profiles and phases of coherent oscillations over time, as demonstrated in the first part of our work, it may provide deeper insight into how seizure-related activity propagates and interacts with the seizure onset zone, offering a more comprehensive understanding of pathological networks and guiding surgical strategies. The second direction focuses on conditions where brain network states change abruptly, such as status epilepticus and its resolution, seizure onset in generalized epilepsy, or the onset of focal status epilepticus. In these scenarios, the dynamics can often be approximated as steady states that transition suddenly. This allows for the derivation of effective models of these steady states, enabling the modeling approach to uncover the temporal and spatial properties of corresponding dominant brain-specific coherent oscillations, as demonstrated in the first part of our work. In addition, incorporating external brain stimulation into the developed brain models, possibly combined with the extensions mentioned above, could produce generative EEG-driven models, based on the results of the second part of our work. These models would allow in-silico testing and optimization of neuromodulative techniques such as transcranial alternating current stimulation (tACS) (Fröhlich, 2015, Polanía et al., 2018), temporal interference stimulation (TIS) (Grossman, 2018) or deep brain stimulation (DBS) (Lozano et al., 2019, Ryvlin et al., 2021)**.** The ultimate goal would be to identify stimulation parameters that effectively transition the brain from pathological to healthy states. Finally, outside epilepsy, alternating steady-state EEG patterns in metabolic, hypoxic, or toxic encephalopaties could also be modeled using the presented approach, to provide quantitative assessments of severity or etiology while distinguishing their oscillatory patterns from epileptic states.

## Author contributions

T.D., L.I. and R.P. conceived the project. T.D. developed the modeling paradigm, and performed the analytical and numerical calculations. L.I and R.P. supervised the project. All authors contributed to the discussion of the results and manuscript writing.

Declaration of generative AI and AI-assisted technologies in the writing process

During the preparation of this work the author(s) used ChatGPT in order to improve language and readability. After using this tool/service, the author(s) reviewed and edited the content as needed and take(s) full responsibility for the content of the publication

## Declaration of competing interest

The authors declare that they have no known competing financial interests or personal relationships that could have appeared to influence the work reported in this paper.
